# Poly[penta-μ-aqua-μ_6_-methyl­ene­disulfonato-μ_5_-methyl­enedisulfonato-tetra­sodium(I)]

**DOI:** 10.1107/S1600536808014037

**Published:** 2008-05-17

**Authors:** Dan-Dan Cao, Zai-Chao Zhang

**Affiliations:** a Department of Chemistry, Huaiyin Teachers College, 111 West Changjiang Road, Huaian 223300, Jiangsu, People’s Republic of China

## Abstract

The title compound, [Na_4_(CH_2_O_6_S_2_)_2_(H_2_O)_5_]_*n*_, was crystallized from an aqueous solution. The sodium ions are surrounded and bridged by O atoms from coordinated water mol­ecules and sulfonate ions in a three-dimensional neutral network. The crystal structure is also stabilized by an intricate system of hydrogen bonds.

## Related literature

The supra­molecular chemistry of the sulfonate group in extended solids constructed by cooperative coordination and other weak inter­molecular inter­actions, as well as the structural and functional properties of Ba^2+^ and Ag^+^ sulfonates, has been reviewed by Côté & Shimizu (2003 ▶). For a review of the structural chemistry and properties of metal arenesulfonates, see: Cai (2004 ▶). For related literature, see: Li *et al.* (2008 ▶); Mi *et al.* (2007 ▶); Videnova-Adrabinska (2007 ▶).
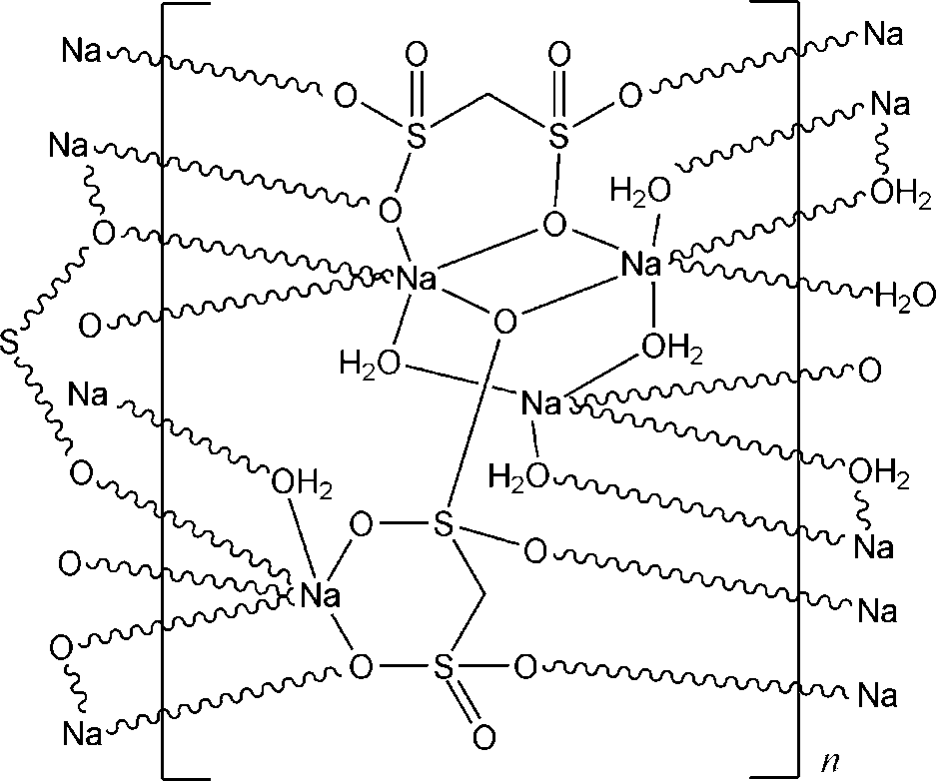


## Experimental

### 

#### Crystal data

[Na_4_(CH_2_O_6_S_2_)_2_(H_2_O)_5_]
*M* *_r_* = 530.33
Triclinic, 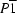

*a* = 8.7758 (7) Å
*b* = 9.5339 (7) Å
*c* = 10.7878 (8) Å
α = 81.425 (2)°
β = 74.545 (2)°
γ = 87.227 (2)°
*V* = 860.19 (11) Å^3^
*Z* = 2
Mo *K*α radiation
μ = 0.74 mm^−1^
*T* = 296 (2) K
0.30 × 0.25 × 0.25 mm

#### Data collection

Bruker SMART APEX2 diffractometer
Absorption correction: multi-scan (*SADABS*; Bruker, 2000 ▶) *T*_min_ = 0.81, *T*_max_ = 0.84
10702 measured reflections
3321 independent reflections
3050 reflections with *I* > 2σ(*I*)
*R*_int_ = 0.022

#### Refinement

*R*[*F* ^2^ > 2σ(*F* ^2^)] = 0.030
*wR*(*F* ^2^) = 0.093
*S* = 1.00
3321 reflections
244 parameters
H-atom parameters constrained
Δρ_max_ = 0.47 e Å^−3^
Δρ_min_ = −0.56 e Å^−3^

### 

Data collection: *APEX2* (Bruker, 2004 ▶); cell refinement: *SAINT* (Bruker, 2004 ▶); data reduction: *SAINT*; program(s) used to solve structure: *SHELXS97* (Sheldrick, 2008 ▶); program(s) used to refine structure: *SHELXL97* (Sheldrick, 2008 ▶); molecular graphics: *ORTEP-3 for Windows* (Farrugia, 1997 ▶); software used to prepare material for publication: *SHELXL97* and *PLATON* (Spek, 2003 ▶).

## Supplementary Material

Crystal structure: contains datablocks global, I. DOI: 10.1107/S1600536808014037/zl2114sup1.cif

Structure factors: contains datablocks I. DOI: 10.1107/S1600536808014037/zl2114Isup2.hkl

Additional supplementary materials:  crystallographic information; 3D view; checkCIF report

## Figures and Tables

**Table 1:** Hydrogen-bond geometry (Å, °)

*D*—H⋯*A*	*D*—H	H⋯*A*	*D*⋯*A*	*D*—H⋯*A*
O13—H13*A*⋯O8^i^	0.97	1.93	2.868 (2)	163
O13—H13*B*⋯O5^ii^	0.97	1.87	2.824 (2)	166
O14—H14*A*⋯O9^iii^	0.97	2.00	2.871 (2)	148
O15—H15*B*⋯O3^iv^	0.97	1.97	2.877 (2)	155
O16—H16*A*⋯O9^iii^	0.97	1.87	2.794 (2)	158
O16—H16*B*⋯O12	0.97	2.18	3.034 (3)	147
O17—H17*A*⋯O4^v^	0.97	2.17	2.879 (3)	129
O17—H17*B*⋯O12^vi^	0.97	2.10	2.918 (3)	141

Symmetry codes: (i) 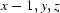; (ii) 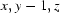; (iii) 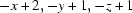; (iv) 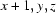; (v) 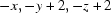; (vi) 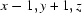.

**Fig. 1.**
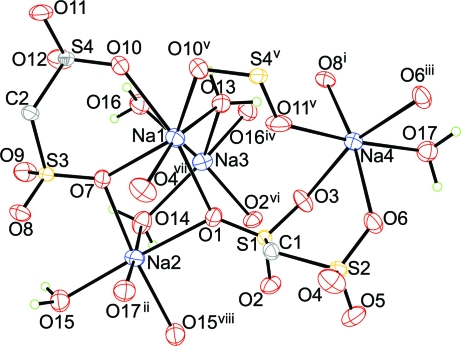
The asymmetric unit of I with atom labels and 50% probability displacement ellipsoids for non-H atoms. H atoms attached to carbon atoms are omitted for clarity. Symmetry codes: (i) -1+x, y, z; (ii) 1+x, y, z; (iii) -x, 2-y, 2-z. (iv) 1-x, 1-y, 1-z; (v) 1-x, 1-y, 2-z; (vi) 1-x, 2-y, 1-z; (vii) 1-x, 2-y, 2-z; (viii) 2-x, 2-y, 1-z;

**Fig. 2.**
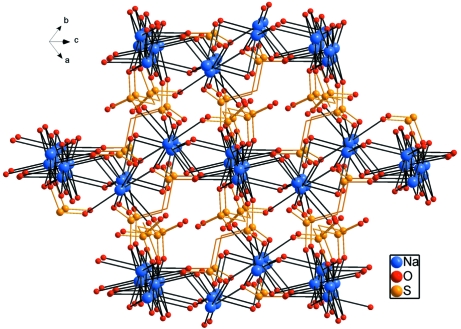
Packing diagram of compound I. Na—O bond is showed by black line, H atoms are omitted for clarity.

## supplementary crystallographic information

### Comment

Due to the weak coordination strength of monosulfonate ions, most metal complexes of these ligands obtained from aqueous solution are water-coordinated metal sulfonate salts, and the coordination chemistry of the sulfonate ion has been less well investigated in comparison with other organic acidato anions such as carbonates and phosfonates (Côté & Shimizu, 2003). However, by employing disulfonates, which can provide multiple potentially chelating coordination sites, stable networks sustained by sulfonate-metal interactions can be obtained with various dimensionalities (Cai, 2004; Li *et al.*, 2008; Mi *et al.*, 2007; Videnova-Adrabinska, 2007).

The present structral study of the title compound {Na_4_(mds)_2_(H_2_O) _5_}*_n_* (I) reveals the existence of sulfonate-sodium interactions. The asymmetric unit consists of four sodium ions, five coordinated *µ*_2_-H_2_O molecules and two CH_3_(SO_3_)_2_^2-^ ligands (Fig. 1). Of the four sodium ions, Na1, Na2 and Na4 are six-coordinated. Na3 is coordinated by five oxygen atoms (O2^vi^, O13, O14, O16 and O16^iv^). The Na—O bond lengths fall in the range of 2.334 (2) to 2.5609 (18) Å. All sulfonate oxygens are deprotonated. Three sulfonate oxygen atoms (O5, O9 and O12) are not coordinated to sodium atoms and do instead act as hydrogen bonding acceptors towards coordinated water molecules. All other hydrogen bonds are from water molecules towards Na coordinated sulfonate oxygen atoms (Table 1).

Each sodium atom is bridged to an equivalent neighbour, forming {Na_2_O_2_} dimers with inversion centers in the middle. O1 and O7 connect the dimers of Na1 and Na2 into an infinite chain. Na1-Na2 chains are joined to dimers of Na3 and Na4 through intricate bridges of oxygens from sulfonate groups and coordinated waters, and O—S—O connectivities, thus generating a three-dimensional network (Fig. 2).

### Experimental

An 1 mol *L*^-1^ NaOH aqueous solution was droped into one of mdsH_2_ (0.14 g, 1 mmol in 20 ml H_2_O) until the pH value of the solution reached 7 to 9. Slow evaporation for several days yieled colorless prismlike crystals of the title compound. Anal. Calcd for C_2_H_14_O_17_Na_4_S_4_ (530.34): C 4.51, H 2.69%; Found: C 4.53, H 2.66%.

### Refinement

All the non-hydrogen atoms were located from the Fourier maps, and were refined anisotropically. H atoms from *µ*_2_-H_2_O can be suitably placed in calculated positions, so all the H atoms, including those attached to carbon atoms and from water molecules, were positioned geometrically, and the isotropic vibration parameters related to the atoms which they are bonded to with U_iso_ = 1.2 U_eq_.

### Crystal data

**Table:**

[Na_4_(CH_2_O_6_S_2_)_2_(H_2_O)_5_]	*Z* = 2
*M**_r_* = 530.33	*F*_000_ = 540
Triclinic, *P*1	*D*_x_ = 2.048 Mg m^−^^3^
Hall symbol: -P 1	Mo *K*α radiation λ = 0.71073 Å
*a* = 8.7758 (7) Å	Cell parameters from 202 reflections
*b* = 9.5339 (7) Å	θ = 2.6–21.1º
*c* = 10.7878 (8) Å	µ = 0.74 mm^−^^1^
α = 81.425 (2)º	*T* = 296 (2) K
β = 74.545 (2)º	Prismlike, colourless
γ = 87.227 (2)º	0.30 × 0.25 × 0.25 mm
*V* = 860.19 (11) Å^3^	

### Data collection

**Table:**

Bruker SMART APEX2 diffractometer	3321 independent reflections
Radiation source: fine-focus sealed tube	3050 reflections with *I* > 2σ(*I*)
Monochromator: graphite	*R*_int_ = 0.022
*T* = 296(2) K	θ_max_ = 26.0º
ω scans	θ_min_ = 2.0º
Absorption correction: multi-scan(SADABS; Bruker, 2000)	*h* = −10→10
*T*_min_ = 0.81, *T*_max_ = 0.84	*k* = −11→11
10702 measured reflections	*l* = −13→13

### Refinement

**Table:**

Refinement on *F*^2^	Hydrogen site location: inferred from neighbouring sites
Least-squares matrix: full	H-atom parameters constrained
*R*[*F*^2^ > 2σ(*F*^2^)] = 0.030	*w* = 1/[σ^2^(*F*_o_^2^) + (0.0428*P*)^2^ + 0.7023*P*] where *P* = (*F*_o_^2^ + 2*F*_c_^2^)/3
*wR*(*F*^2^) = 0.093	(Δ/σ)_max_ = 0.001
*S* = 1.00	Δρ_max_ = 0.47 e Å^−^^3^
3321 reflections	Δρ_min_ = −0.56 e Å^−^^3^
244 parameters	Extinction correction: SHELXL97 (Sheldrick, 2008), F*=F(1+0.002xF^2^(sin(2θ))^-0.25^
Primary atom site location: structure-invariant direct methods	Extinction coefficient: 0.00025 (5)
Secondary atom site location: difference Fourier map	

### Special details

**Table:**

Geometry. All esds (except the esd in the dihedral angle between two l.s. planes) are estimated using the full covariance matrix. The cell esds are taken into account individually in the estimation of esds in distances, angles and torsion angles; correlations between esds in cell parameters are only used when they are defined by crystal symmetry. An approximate (isotropic) treatment of cell esds is used for estimating esds involving l.s. planes.
Refinement. Refinement of *F*^2^ against ALL reflections. The weighted *R*-factor *wR* and goodness of fit *S* are based on *F*^2^, conventional *R*-factors *R* are based on *F*, with *F* set to zero for negative *F*^2^. The threshold expression of *F*^2^ > σ(*F*^2^) is used only for calculating *R*-factors(gt) *etc*. and is not relevant to the choice of reflections for refinement. *R*-factors based on *F*^2^ are statistically about twice as large as those based on *F*, and *R*- factors based on ALL data will be even larger.

### Fractional atomic coordinates and isotropic or equivalent isotropic displacement parameters (Å^2^)

**Table:**

	*x*	*y*	*z*	*U*_iso_*/*U*_eq_	
C1	0.4436 (2)	1.0561 (2)	0.8451 (2)	0.0189 (4)	
H1A	0.4297	0.9743	0.9121	0.023*	
H1B	0.5440	1.0983	0.8394	0.023*	
C2	0.9094 (3)	0.4391 (2)	0.8842 (2)	0.0217 (4)	
H2A	1.0010	0.3786	0.8863	0.026*	
H2B	0.8798	0.4796	0.9647	0.026*	
Na1	0.55562 (10)	0.66480 (9)	0.86815 (9)	0.0253 (2)	
Na2	0.85901 (11)	0.87496 (9)	0.59357 (9)	0.0268 (2)	
Na3	0.54875 (13)	0.67712 (11)	0.52620 (12)	0.0421 (3)	
Na4	0.08149 (10)	0.88263 (9)	0.88189 (8)	0.0240 (2)	
O1	0.57149 (19)	0.87897 (16)	0.69116 (15)	0.0261 (4)	
O2	0.5103 (2)	1.11297 (17)	0.59433 (15)	0.0278 (4)	
O3	0.29960 (18)	0.94639 (19)	0.70181 (15)	0.0287 (4)	
O4	0.3169 (2)	1.21026 (19)	1.01825 (16)	0.0334 (4)	
O5	0.3122 (2)	1.30241 (17)	0.79628 (18)	0.0353 (4)	
O6	0.13991 (19)	1.11171 (17)	0.91904 (17)	0.0290 (4)	
O7	0.82527 (18)	0.65404 (17)	0.73482 (15)	0.0259 (4)	
O8	1.06688 (19)	0.66657 (17)	0.80084 (17)	0.0286 (4)	
O9	1.0542 (2)	0.51641 (18)	0.64174 (16)	0.0325 (4)	
O10	0.6110 (2)	0.41828 (18)	0.9018 (2)	0.0366 (4)	
O11	0.7435 (2)	0.21996 (18)	0.99171 (18)	0.0352 (4)	
O12	0.7916 (2)	0.27615 (19)	0.75861 (17)	0.0367 (4)	
O13	0.3962 (2)	0.59164 (17)	0.74448 (17)	0.0316 (4)	
H13A	0.2921	0.6356	0.7642	0.038*	
H13B	0.3854	0.4893	0.7581	0.038*	
O14	0.8268 (2)	0.7545 (2)	0.42866 (17)	0.0361 (4)	
H14A	0.8993	0.6746	0.4159	0.043*	
H14B	0.8388	0.8170	0.3470	0.043*	
O15	1.1401 (2)	0.88559 (19)	0.51526 (17)	0.0338 (4)	
H15A	1.1832	0.8177	0.4560	0.041*	
H15B	1.1927	0.8759	0.5846	0.041*	
O16	0.6570 (2)	0.43211 (19)	0.54436 (17)	0.0355 (4)	
H16A	0.7614	0.4243	0.4860	0.043*	
H16B	0.6587	0.3945	0.6326	0.043*	
O17	−0.1158 (2)	0.97729 (18)	0.77543 (16)	0.0308 (4)	
H17A	−0.2173	0.9697	0.8396	0.037*	
H17B	−0.0951	1.0776	0.7471	0.037*	
S1	0.45658 (6)	0.99467 (5)	0.69417 (5)	0.01723 (13)	
S2	0.29091 (6)	1.18145 (5)	0.89699 (5)	0.02035 (14)	
S3	0.96766 (6)	0.58056 (5)	0.75349 (5)	0.01822 (14)	
S4	0.75141 (6)	0.33016 (5)	0.88177 (5)	0.01945 (14)	

### Atomic displacement parameters (Å^2^)

**Table:**

	*U*^11^	*U*^22^	*U*^33^	*U*^12^	*U*^13^	*U*^23^
C1	0.0163 (10)	0.0213 (10)	0.0196 (9)	−0.0008 (8)	−0.0062 (8)	−0.0015 (8)
C2	0.0201 (11)	0.0206 (10)	0.0249 (10)	−0.0059 (8)	−0.0097 (9)	0.0031 (8)
Na1	0.0236 (5)	0.0240 (5)	0.0298 (5)	−0.0020 (3)	−0.0086 (4)	−0.0049 (4)
Na2	0.0269 (5)	0.0246 (5)	0.0278 (5)	−0.0050 (4)	−0.0061 (4)	−0.0010 (4)
Na3	0.0352 (6)	0.0290 (5)	0.0592 (7)	−0.0041 (4)	−0.0151 (5)	0.0090 (5)
Na4	0.0222 (5)	0.0225 (4)	0.0275 (4)	−0.0026 (3)	−0.0064 (4)	−0.0035 (3)
O1	0.0236 (8)	0.0227 (8)	0.0298 (8)	0.0048 (6)	−0.0047 (6)	−0.0026 (6)
O2	0.0333 (9)	0.0226 (8)	0.0217 (8)	0.0005 (7)	−0.0009 (7)	0.0034 (6)
O3	0.0179 (8)	0.0449 (10)	0.0253 (8)	−0.0059 (7)	−0.0055 (6)	−0.0102 (7)
O4	0.0336 (10)	0.0408 (10)	0.0289 (9)	−0.0089 (8)	−0.0056 (7)	−0.0161 (8)
O5	0.0451 (11)	0.0193 (8)	0.0410 (10)	0.0027 (7)	−0.0139 (8)	0.0013 (7)
O6	0.0177 (8)	0.0302 (9)	0.0412 (9)	−0.0024 (7)	−0.0055 (7)	−0.0149 (7)
O7	0.0199 (8)	0.0258 (8)	0.0296 (8)	0.0012 (6)	−0.0064 (6)	0.0031 (6)
O8	0.0241 (8)	0.0224 (8)	0.0401 (9)	−0.0078 (6)	−0.0080 (7)	−0.0050 (7)
O9	0.0320 (9)	0.0296 (9)	0.0299 (9)	−0.0016 (7)	0.0049 (7)	−0.0088 (7)
O10	0.0195 (8)	0.0249 (9)	0.0626 (12)	−0.0013 (7)	−0.0085 (8)	−0.0010 (8)
O11	0.0371 (10)	0.0292 (9)	0.0376 (9)	−0.0137 (8)	−0.0151 (8)	0.0151 (7)
O12	0.0471 (11)	0.0332 (10)	0.0309 (9)	−0.0113 (8)	−0.0073 (8)	−0.0097 (7)
O13	0.0273 (9)	0.0237 (8)	0.0458 (10)	−0.0008 (7)	−0.0128 (8)	−0.0051 (7)
O14	0.0411 (11)	0.0366 (10)	0.0339 (9)	0.0084 (8)	−0.0144 (8)	−0.0096 (8)
O15	0.0280 (9)	0.0371 (10)	0.0331 (9)	0.0018 (7)	−0.0063 (7)	0.0011 (7)
O16	0.0296 (9)	0.0405 (10)	0.0330 (9)	−0.0025 (8)	−0.0060 (7)	0.0020 (8)
O17	0.0318 (9)	0.0290 (9)	0.0328 (9)	−0.0004 (7)	−0.0097 (7)	−0.0061 (7)
S1	0.0149 (3)	0.0180 (3)	0.0175 (2)	−0.00013 (19)	−0.00291 (19)	−0.00080 (19)
S2	0.0192 (3)	0.0183 (3)	0.0247 (3)	−0.0022 (2)	−0.0057 (2)	−0.0062 (2)
S3	0.0165 (3)	0.0157 (3)	0.0210 (3)	−0.00201 (19)	−0.00272 (19)	−0.00110 (19)
S4	0.0192 (3)	0.0153 (3)	0.0240 (3)	−0.00369 (19)	−0.0073 (2)	0.00080 (19)

### Geometric parameters (Å, °)

**Table:**

C1—S2	1.783 (2)	Na4—O17	2.3942 (19)
C1—S1	1.785 (2)	Na4—O11^ii^	2.3972 (19)
C1—H1A	0.9700	Na4—O6^viii^	2.4881 (18)
C1—H1B	0.9700	Na4—Na4^viii^	3.6050 (17)
C2—S4	1.780 (2)	Na4—Na2^vii^	4.1000 (13)
C2—S3	1.785 (2)	O1—S1	1.4562 (16)
C2—H2A	0.9700	O2—S1	1.4398 (16)
C2—H2B	0.9700	O2—Na3^v^	2.3339 (18)
Na1—O4^i^	2.3382 (19)	O3—S1	1.4512 (16)
Na1—O13	2.3644 (19)	O4—S2	1.4528 (17)
Na1—O10	2.3709 (19)	O4—Na1^i^	2.3382 (19)
Na1—O7	2.4260 (18)	O5—S2	1.4437 (18)
Na1—O10^ii^	2.552 (2)	O6—S2	1.4577 (17)
Na1—O1	2.5607 (18)	O6—Na4^viii^	2.4881 (18)
Na1—O11^ii^	2.913 (2)	O7—S3	1.4492 (16)
Na1—S4^ii^	3.2699 (10)	O8—S3	1.4569 (16)
Na1—Na3	3.6898 (15)	O8—Na4^iii^	2.3763 (18)
Na1—Na2	3.7975 (13)	O9—S3	1.4460 (16)
Na1—Na1^ii^	3.8822 (18)	O10—S4	1.4432 (17)
Na2—O14	2.3389 (19)	O10—Na1^ii^	2.552 (2)
Na2—O17^iii^	2.3816 (19)	O11—S4	1.4515 (17)
Na2—O15	2.3866 (19)	O11—Na4^ii^	2.3972 (19)
Na2—O7	2.3893 (18)	O11—Na1^ii^	2.913 (2)
Na2—O15^iv^	2.4057 (19)	O12—S4	1.4462 (17)
Na2—O1	2.4623 (18)	O13—H13A	0.9700
Na2—Na2^iv^	3.5152 (18)	O13—H13B	0.9700
Na2—Na3	3.6688 (15)	O14—H14A	0.9700
Na2—Na4^iii^	4.1000 (13)	O14—H14B	0.9700
Na3—O2^v^	2.3339 (18)	O15—Na2^iv^	2.4057 (19)
Na3—O13	2.420 (2)	O15—H15A	0.9700
Na3—O16^vi^	2.465 (2)	O15—H15B	0.9700
Na3—O16	2.479 (2)	O16—Na3^vi^	2.465 (2)
Na3—O14	2.486 (2)	O16—H16A	0.9700
Na3—O1	2.856 (2)	O16—H16B	0.9700
Na3—Na3^vi^	3.683 (2)	O17—Na2^vii^	2.3816 (19)
Na4—O3	2.3635 (18)	O17—H17A	0.9700
Na4—O8^vii^	2.3763 (18)	O17—H17B	0.9700
Na4—O6	2.3816 (18)	S4—Na1^ii^	3.2699 (10)
			
S2—C1—S1	117.32 (11)	O13—Na3—Na2	100.89 (6)
S2—C1—H1A	108.0	O16^vi^—Na3—Na2	172.47 (6)
S1—C1—H1A	108.0	O16—Na3—Na2	101.22 (6)
S2—C1—H1B	108.0	O14—Na3—Na2	39.03 (5)
S1—C1—H1B	108.0	O1—Na3—Na2	42.08 (4)
H1A—C1—H1B	107.2	O2^v^—Na3—Na3^vi^	123.29 (7)
S4—C2—S3	117.32 (12)	O13—Na3—Na3^vi^	81.18 (5)
S4—C2—H2A	108.0	O16^vi^—Na3—Na3^vi^	41.99 (5)
S3—C2—H2A	108.0	O16—Na3—Na3^vi^	41.70 (5)
S4—C2—H2B	108.0	O14—Na3—Na3^vi^	116.43 (7)
S3—C2—H2B	108.0	O1—Na3—Na3^vi^	151.94 (6)
H2A—C2—H2B	107.2	Na2—Na3—Na3^vi^	142.60 (5)
O4^i^—Na1—O13	166.18 (8)	O2^v^—Na3—Na1	121.20 (6)
O4^i^—Na1—O10	111.23 (8)	O13—Na3—Na1	38.99 (5)
O13—Na1—O10	82.42 (7)	O16^vi^—Na3—Na1	124.37 (6)
O4^i^—Na1—O7	80.23 (6)	O16—Na3—Na1	85.67 (5)
O13—Na1—O7	105.45 (7)	O14—Na3—Na1	96.42 (5)
O10—Na1—O7	78.58 (6)	O1—Na3—Na1	43.79 (4)
O4^i^—Na1—O10^ii^	81.40 (7)	Na2—Na3—Na1	62.14 (3)
O13—Na1—O10^ii^	100.65 (7)	Na3^vi^—Na3—Na1	109.09 (4)
O10—Na1—O10^ii^	75.97 (7)	O3—Na4—O8^vii^	88.53 (6)
O7—Na1—O10^ii^	140.45 (7)	O3—Na4—O6	78.83 (6)
O4^i^—Na1—O1	91.18 (7)	O8^vii^—Na4—O6	167.28 (7)
O13—Na1—O1	78.18 (6)	O3—Na4—O17	95.65 (7)
O10—Na1—O1	142.97 (7)	O8^vii^—Na4—O17	87.86 (6)
O7—Na1—O1	76.66 (6)	O6—Na4—O17	92.01 (6)
O10^ii^—Na1—O1	138.45 (6)	O3—Na4—O11^ii^	90.43 (7)
O4^i^—Na1—O11^ii^	88.36 (6)	O8^vii^—Na4—O11^ii^	92.18 (7)
O13—Na1—O11^ii^	82.51 (6)	O6—Na4—O11^ii^	89.29 (7)
O10—Na1—O11^ii^	120.57 (6)	O17—Na4—O11^ii^	173.92 (7)
O7—Na1—O11^ii^	160.46 (6)	O3—Na4—O6^viii^	163.22 (7)
O10^ii^—Na1—O11^ii^	51.33 (5)	O8^vii^—Na4—O6^viii^	108.07 (6)
O1—Na1—O11^ii^	87.86 (5)	O6—Na4—O6^viii^	84.51 (6)
O4^i^—Na1—S4^ii^	85.44 (5)	O17—Na4—O6^viii^	82.85 (6)
O13—Na1—S4^ii^	90.84 (5)	O11^ii^—Na4—O6^viii^	91.36 (7)
O10—Na1—S4^ii^	97.33 (5)	O3—Na4—Na4^viii^	122.20 (6)
O7—Na1—S4^ii^	162.36 (5)	O8^vii^—Na4—Na4^viii^	149.15 (6)
O10^ii^—Na1—S4^ii^	25.03 (4)	O6—Na4—Na4^viii^	43.39 (4)
O1—Na1—S4^ii^	114.07 (5)	O17—Na4—Na4^viii^	86.40 (5)
O11^ii^—Na1—S4^ii^	26.35 (3)	O11^ii^—Na4—Na4^viii^	90.47 (6)
O4^i^—Na1—Na3	135.93 (6)	O6^viii^—Na4—Na4^viii^	41.12 (4)
O13—Na1—Na3	40.08 (5)	O3—Na4—Na2^vii^	81.93 (5)
O10—Na1—Na3	95.38 (6)	O8^vii^—Na4—Na2^vii^	60.20 (5)
O7—Na1—Na3	71.14 (5)	O6—Na4—Na2^vii^	115.90 (5)
O10^ii^—Na1—Na3	140.70 (6)	O17—Na4—Na2^vii^	30.76 (4)
O1—Na1—Na3	50.52 (4)	O11^ii^—Na4—Na2^vii^	151.34 (6)
O11^ii^—Na1—Na3	108.04 (5)	O6^viii^—Na4—Na2^vii^	103.86 (5)
S4^ii^—Na1—Na3	126.48 (3)	Na4^viii^—Na4—Na2^vii^	117.00 (4)
O4^i^—Na1—Na2	78.15 (5)	S1—O1—Na2	131.01 (10)
O13—Na1—Na2	98.51 (5)	S1—O1—Na1	125.55 (9)
O10—Na1—Na2	114.45 (6)	Na2—O1—Na1	98.21 (6)
O7—Na1—Na2	37.60 (4)	S1—O1—Na3	114.45 (9)
O10^ii^—Na1—Na2	159.32 (5)	Na2—O1—Na3	86.90 (5)
O1—Na1—Na2	39.92 (4)	Na1—O1—Na3	85.68 (5)
O11^ii^—Na1—Na2	124.52 (4)	S1—O2—Na3^v^	148.85 (11)
S4^ii^—Na1—Na2	147.72 (3)	S1—O3—Na4	131.30 (9)
Na3—Na1—Na2	58.66 (3)	S2—O4—Na1^i^	150.62 (11)
O4^i^—Na1—Na1^ii^	97.06 (6)	S2—O6—Na4	130.70 (10)
O13—Na1—Na1^ii^	92.35 (5)	S2—O6—Na4^viii^	123.77 (9)
O10—Na1—Na1^ii^	39.63 (5)	Na4—O6—Na4^viii^	95.49 (6)
O7—Na1—Na1^ii^	112.71 (5)	S3—O7—Na2	116.86 (9)
O10^ii^—Na1—Na1^ii^	36.33 (4)	S3—O7—Na1	134.13 (9)
O1—Na1—Na1^ii^	168.41 (6)	Na2—O7—Na1	104.11 (6)
O11^ii^—Na1—Na1^ii^	84.25 (4)	S3—O8—Na4^iii^	144.64 (11)
S4^ii^—Na1—Na1^ii^	58.81 (2)	S4—O10—Na1	135.88 (11)
Na3—Na1—Na1^ii^	124.47 (4)	S4—O10—Na1^ii^	106.52 (10)
Na2—Na1—Na1^ii^	150.21 (4)	Na1—O10—Na1^ii^	104.03 (7)
O14—Na2—O17^iii^	174.53 (8)	S4—O11—Na4^ii^	136.83 (11)
O14—Na2—O15	94.48 (7)	S4—O11—Na1^ii^	90.67 (9)
O17^iii^—Na2—O15	87.48 (7)	Na4^ii^—O11—Na1^ii^	117.01 (7)
O14—Na2—O7	88.36 (7)	Na1—O13—Na3	100.93 (7)
O17^iii^—Na2—O7	86.24 (6)	Na1—O13—H13A	111.6
O15—Na2—O7	101.40 (7)	Na3—O13—H13A	111.6
O14—Na2—O15^iv^	99.06 (7)	Na1—O13—H13B	111.6
O17^iii^—Na2—O15^iv^	86.17 (7)	Na3—O13—H13B	111.6
O15—Na2—O15^iv^	85.64 (7)	H13A—O13—H13B	109.4
O7—Na2—O15^iv^	169.40 (7)	Na2—O14—Na3	98.94 (7)
O14—Na2—O1	91.38 (7)	Na2—O14—H14A	112.0
O17^iii^—Na2—O1	86.73 (6)	Na3—O14—H14A	112.0
O15—Na2—O1	174.12 (7)	Na2—O14—H14B	112.0
O7—Na2—O1	79.24 (6)	Na3—O14—H14B	112.0
O15^iv^—Na2—O1	92.95 (6)	H14A—O14—H14B	109.7
O14—Na2—Na2^iv^	99.26 (6)	Na2—O15—Na2^iv^	94.36 (7)
O17^iii^—Na2—Na2^iv^	85.67 (6)	Na2—O15—H15A	112.9
O15—Na2—Na2^iv^	43.03 (5)	Na2^iv^—O15—H15A	112.9
O7—Na2—Na2^iv^	143.79 (6)	Na2—O15—H15B	112.9
O15^iv^—Na2—Na2^iv^	42.61 (5)	Na2^iv^—O15—H15B	112.9
O1—Na2—Na2^iv^	135.28 (6)	H15A—O15—H15B	110.3
O14—Na2—Na3	42.03 (5)	Na3^vi^—O16—Na3	96.30 (7)
O17^iii^—Na2—Na3	134.64 (6)	Na3^vi^—O16—H16A	112.5
O15—Na2—Na3	134.80 (6)	Na3—O16—H16A	112.5
O7—Na2—Na3	71.87 (5)	Na3^vi^—O16—H16B	112.5
O15^iv^—Na2—Na3	108.79 (6)	Na3—O16—H16B	112.5
O1—Na2—Na3	51.02 (5)	H16A—O16—H16B	110.0
Na2^iv^—Na2—Na3	134.34 (4)	Na2^vii^—O17—Na4	118.30 (8)
O14—Na2—Na1	96.33 (6)	Na2^vii^—O17—H17A	107.7
O17^iii^—Na2—Na1	78.85 (5)	Na4—O17—H17A	107.7
O15—Na2—Na1	137.49 (5)	Na2^vii^—O17—H17B	107.7
O7—Na2—Na1	38.29 (4)	Na4—O17—H17B	107.7
O15^iv^—Na2—Na1	132.48 (5)	H17A—O17—H17B	107.1
O1—Na2—Na1	41.87 (4)	O2—S1—O3	113.99 (10)
Na2^iv^—Na2—Na1	164.30 (4)	O2—S1—O1	113.19 (9)
Na3—Na2—Na1	59.20 (3)	O3—S1—O1	112.55 (10)
O14—Na2—Na4^iii^	146.42 (6)	O2—S1—C1	106.32 (10)
O17^iii^—Na2—Na4^iii^	30.94 (5)	O3—S1—C1	106.12 (9)
O15—Na2—Na4^iii^	66.79 (5)	O1—S1—C1	103.62 (10)
O7—Na2—Na4^iii^	69.72 (5)	O5—S2—O4	114.42 (11)
O15^iv^—Na2—Na4^iii^	106.56 (5)	O5—S2—O6	112.59 (11)
O1—Na2—Na4^iii^	108.29 (5)	O4—S2—O6	111.20 (10)
Na2^iv^—Na2—Na4^iii^	85.84 (3)	O5—S2—C1	107.49 (10)
Na3—Na2—Na4^iii^	139.39 (3)	O4—S2—C1	102.79 (10)
Na1—Na2—Na4^iii^	82.22 (3)	O6—S2—C1	107.57 (10)
O2^v^—Na3—O13	123.40 (7)	O9—S3—O7	113.00 (10)
O2^v^—Na3—O16^vi^	86.03 (7)	O9—S3—O8	112.97 (10)
O13—Na3—O16^vi^	85.41 (7)	O7—S3—O8	112.99 (10)
O2^v^—Na3—O16	152.16 (8)	O9—S3—C2	106.55 (10)
O13—Na3—O16	81.48 (6)	O7—S3—C2	107.55 (10)
O16^vi^—Na3—O16	83.70 (7)	O8—S3—C2	102.91 (10)
O2^v^—Na3—O14	83.66 (7)	O10—S4—O12	113.60 (12)
O13—Na3—O14	134.23 (8)	O10—S4—O11	111.27 (11)
O16^vi^—Na3—O14	136.85 (8)	O12—S4—O11	113.07 (11)
O16—Na3—O14	86.32 (7)	O10—S4—C2	106.45 (10)
O2^v^—Na3—O1	79.21 (6)	O12—S4—C2	107.98 (11)
O13—Na3—O1	71.68 (6)	O11—S4—C2	103.70 (10)
O16^vi^—Na3—O1	138.69 (7)	O10—S4—Na1^ii^	48.45 (8)
O16—Na3—O1	124.33 (7)	O12—S4—Na1^ii^	137.74 (8)
O14—Na3—O1	79.74 (6)	O11—S4—Na1^ii^	62.98 (8)
O2^v^—Na3—Na2	87.04 (5)	C2—S4—Na1^ii^	113.81 (8)

Symmetry codes: (i) −*x*+1, −*y*+2, −*z*+2; (ii) −*x*+1, −*y*+1, −*z*+2; (iii) *x*+1, *y*, *z*; (iv) −*x*+2, −*y*+2, −*z*+1; (v) −*x*+1, −*y*+2, −*z*+1; (vi) −*x*+1, −*y*+1, −*z*+1; (vii) *x*−1, *y*, *z*; (viii) −*x*, −*y*+2, −*z*+2.

### Hydrogen-bond geometry (Å, °)

**Table:**

*D*—H···*A*	*D*—H	H···*A*	*D*···*A*	*D*—H···*A*
O13—H13A···O8^vii^	0.97	1.93	2.868 (2)	163
O13—H13B···O5^ix^	0.97	1.87	2.824 (2)	166
O14—H14A···O9^x^	0.97	2.00	2.871 (2)	148
O15—H15B···O3^iii^	0.97	1.97	2.877 (2)	155
O16—H16A···O9^x^	0.97	1.87	2.794 (2)	158
O16—H16B···O12	0.97	2.18	3.034 (3)	147
O17—H17A···O4^viii^	0.97	2.17	2.879 (3)	129
O17—H17B···O12^xi^	0.97	2.10	2.918 (3)	141

Symmetry codes: (vii) *x*−1, *y*, *z*; (ix) *x*, *y*−1, *z*; (x) −*x*+2, −*y*+1, −*z*+1; (iii) *x*+1, *y*, *z*; (viii) −*x*, −*y*+2, −*z*+2; (xi) *x*−1, *y*+1, *z*.
